# Evaluation of the Modification Effects of Heparin/Dalteparin on Silk Fibroin Structure and Physical Properties for Skin Wound Healing

**DOI:** 10.3390/polym16030321

**Published:** 2024-01-24

**Authors:** Rikako Hama, Yasumoto Nakazawa

**Affiliations:** Department of Biotechnology and Life Science, Graduate School of Engineering, Tokyo University of Agriculture and Technology, 2-24-16 Naka-Cho, Koganei 184-8588, Japan; rikako-hama@st.go.tuat.ac.jp

**Keywords:** silk fibroin, heparin, tissue engineering, wound healing, dressing material

## Abstract

We have developed a functionalized silk fibroin (BSF) that can serve as an improved fundamental material for dressings by specifically capturing growth factors secreted during the healing process and supplying them to cells accumulated in the wound area to enhance the tissue regeneration efficiency. When considering the design of heparin-modified BSF, there is a difficulty with binding to high-molecular-weight polysaccharides without disrupting the hydrophobic crystalline structure of the BSF. In this study, a low-molecular-weight pharmaceutical heparin, dalteparin, was selected and cross-linked with the tyrosine residue presence in the BSF non-crystalline region. When targeting 3D porous applications like nanofiber sheets, as it is crucial not only to enhance biological activity but also to improve handling by maintaining stability in water and mechanical strength, a trade-off between improved cell affinity and reduced mechanical strength depending on crystalline structure was evaluated. The use of dalteparin maintained the mechanical strength better than unfractionated heparin by reducing the effect on disturbing BSF recrystallization. Film surface hydrophilicity and cell proliferation induction were significantly higher in the dalteparin group. For BSF functionalization, using purified heparin was an effective approach that achieved a balance between preserving the mechanical properties and induction of tissue regeneration, offering the potential for various forms in the future.

## 1. Introduction

Skin wounds are caused by a variety of factors, including trauma, physical defects such as surgery, diabetes, and pressure ulcers. Skin tissue acts as a barrier between the body and the outside world and has originally excellent repair mechanisms. In cases in which repair of significant damage exceeds the patient’s regenerative capacity, wound dressings are used to restore neotissue with function and aesthetics. In cases of extensive skin defects and chronic wounds (e.g., ulcers and diabetes), delayed tissue regeneration poses a significant challenge. Achieving prompt epithelialization is crucial to prevent prolonged inflammation and secondary wound infections [1]. This necessitates active tissue neogenesis during the proliferative phase following the stage of the inflammatory response. As the healing process becomes clearer, the role of wound dressings is changing to the acquisition of native-like regenerative tissue. In addition to providing a moist healing environment and protection from secondary external stimuli, it is required to restore the skin’s natural biological functions. Tissue engineering studies have been conducted to provide rapid new tissue formation and maturation [2,3]. Tissue engineering materials are designed by focusing on the relationships among three elements: cells, scaffolds, and growth factors (GFs). For severe burns and other difficult-to-heal wounds, the use of cultured epidermal sheets, prepared by culturing cells collected from patients, and materials containing large amounts of cultured cells and artificial GFs is a useful approach [4,5]. However, cell culture has risks, such as increased material fabrication time and cost and contamination by other biogenic substances in the culture medium. Peptides fabricated from GFs or their functional sequences promote epithelialization and granulation tissue formation and shorten healing time, but their diffusion from the material and low stability in vivo reduce their cost-effectiveness [6]. The increasing number of patients with potential skin injuries in today’s society requires the development of more versatile, low-cost wound dressings [7].

We have investigated a material concept that specifically captures GF secreted during the healing process and supplies it to cells accumulated in the wound site, thereby increasing the efficiency of tissue regeneration [8]. This is expected to maximize the effectiveness of the noninvasive and versatile material based on the healing mechanism of the living body. We focused on heparin, which is a component of the extracellular matrix (ECM). Heparin forms complexes with multiple GFs secreted in the exudate through electrostatic interactions and works to regulate cellular functions such as proliferation, differentiation, and migration by promoting binding to GF receptors on the cell membrane [7,9,10,11,12]: vascular endothelial growth factor (VEGF), fibroblast growth factor (FGF), and epidermal growth factor (EGF). Silk fibroin (BSF), which is biodegradable, low-inflammatory, and capable of promoting skin regeneration and fibroblast proliferation, was selected as the base material to hold these functional molecules in the material [13,14,15]. BSF can be easily converted to aqueous solution by dissolution in concentrated salt solutions such as LiBr and dialysis, and it can be processed into various forms such as films, hydrogels, and nanofiber sheets [16,17,18,19]. BSF forms a hydrophobic crystal structure with a densely aggregated β-sheet structure. Therefore, the degradation period and mechanical strength of the recycled material after molding and processing depend on the state of the reformed crystal structure. The β-sheet structure is composed of physical cross-links between repeating sequences (Gly-Ala-Gly-Ala-Gly-Ser)_n_ present in the amino acid sequence, interspersed with amorphous portions consisting of sequences substituted with polar, bulky side chains such as Tyr residues [20].

Studies on the combination of heparin and BSF have been reported. A nonwoven nanofiber sheet was produced by collecting BSF/formic acid solution into a collector filled with heparin aqueous solution [21]. The fiber surface being covered with heparin improved blood compatibility, and the proliferation of Vero cells was not inhibited. In another study, a two-layered material was developed, consisting of a BSF sponge chemically modified with heparin using the EDC/NHS chemical reaction and BSF nanofibers containing silver sulfadiazine [22]. The presence of silver inhibited bacterial growth. It also exhibited a high water content of up to eight times, equivalent to existing cover materials. In the development of biomaterials, it is important to understand cell responses and the maturity of newly formed tissues in comparison with native skin tissue. Thus, the combination of BSF as a foundation and heparin as a functional molecule has shown effectiveness in inducing skin tissue regeneration.

Each action in tissue regeneration arises from the material characters; therefore, it is desirable to control the properties through fabricating methods that offer higher reproducibility and uniformity. However, as is common with other biomaterials, there is a challenge with the difficulty of optimizing material characteristics through further fine-tuning due to the inherent heterogeneity and processing instability of the material. A chemical modification method was employed to prevent short-term loss of heparin from the material. When applying a hydrogel composed of polyethylene glycol (PEG) and dithiothreitol supplemented with heparin and FGF to rat skin defects, wound closure and tissue regeneration were improved [23]. With the swelling of the hydrogel, an initial loss of both heparin and FGF was observed, but the sustained release over a period of 10 days appeared beneficial for wound closure and the acquisition of regenerating epithelium. BSF can control the degradation period through its crystallinity. By maintaining the heparin–GF complex in the material, it is expected to establish a higher binding affinity to cell receptors during the period that adequately covers the proliferation phase, compared with simply allowing sustained release. 

In our previous study, the chemical modification of unfractionated heparin to Tyr residues did not inhibit the recrystallization of BSF, but achieved secondary acquisition of water-containing capacity due to the partially disrupted β-sheet structure [8]. On the other hand, the water-containing capacity and skin fibroblast proliferation results showed the same level above a certain amount of modification. Here is a trade-off between the improvement of cell affinity and the decrease in mechanical strength in the water-containing state due to disruption of the crystal structure. As a polysaccharide, heparin has a wide molecular weight distribution and branching structure due to irregular sugar repeats, making it difficult to organize the relationship. Heparin also has been utilized for its anticoagulant properties, for postoperative intravenous administration to patients, and for coating materials such as artificial blood vessels at the blood–material interface [24,25]. To reduce the side effects related to bleeding, low-molecular-weight heparin has been employed. Therefore, we newly recruited dalteparin, a drug product derived from porcine-derived heparin that is nitrite-degraded and depolymerized to an average molecular weight of approximately 5000–6000 [26]. Dalteparin maintains its inhibitory effect on factor Xa through antithrombin III. Furthermore, continuous subcutaneous administration of dalteparin has been shown to promote angiogenesis through binding with VEGF [26]. In addition, as a method for chemically modifying the reactive amino acid side chains of BSF, commonly chosen approaches include 1-ethyl-3-(3-dimethylaminopropyl) carbodiimide (EDC)/N-hydroxysuccinimide (NHS) crosslinking [27], utilization of cyanuric chloride (CY) as a crosslinker [28], and methacrylation [29]. Due to considerations of the ease of material safety verification and simplicity, CY, which can selectively bind to tyrosine residues in the amorphous region, was employed. Unfractionated heparin (Hep-High) and low-molecular-weight dalteparin (Hep-Low) were chemically modified to BSF with different modification amounts in a stepwise manner, and the structure, physical properties, and cell affinity of the resulting regenerated films were evaluated. The effects of decreasing molecular weight on cell proliferation and recrystallization were compared and discussed. In addition, for future application to three-dimensional porous materials, it is necessary to select an appropriate modification amount in consideration of the material’s ease of handling. The relationship between the regenerated crystal structure emphasized by changing the modification amount and its effect on physical properties, together with the cell growth effect, was discussed to obtain an index for the modification amount. 

## 2. Materials and Methods

### 2.1. Preparation of BSF Aqueous Solution

Silk fibers were obtained from *Bombyx mori* cocoons by reeling, as previously outlined [30]. Then, 250 g of dry silk fibers were placed in a 0.02 M sodium carbonate aqueous solution at 95 °C for 30 min to degum the sericin. Fibers were rinsed with water at 40 °C several times and dried at room temperature to obtain the BSF fibers. The degummed BSF fibers were dissolved in 9 M aqueous lithium bromide (LiBr) at 37 °C for 2 h. The BSF/LiBr aqueous solution was dialyzed in cellulose membrane (MWCO, 12,000–14,000, ASONE, Osaka, Japan) against ultrapure water until the electric conductivity ceased to decrease, followed by centrifugation. The final concentration of the purified BSF aqueous solution was approximately 4 wt%. To obtain BSF sponge, the solution was diluted to 1 wt% and lyophilized.

### 2.2. Chemical Modification of BSF with Heparin

**Figure 1:**
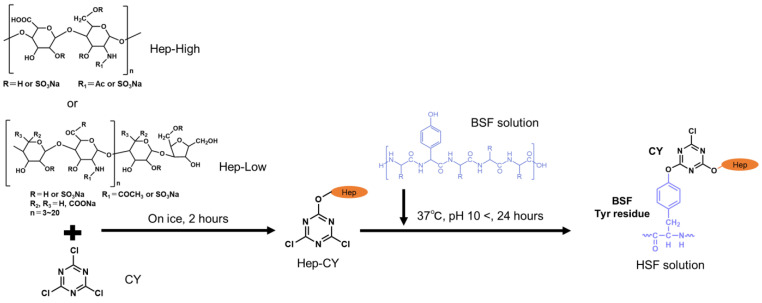
A scheme of the chemical modification reaction of heparin-modified silk fibroin (HSF). Silk fibroin (BSF) and heparin (Hep-High) or dalteparin (Hep-Low) were cross-linked via cyanuric chloride (CY). Side chains are R = H or SO_3_Na, R1 = Ac or SO_3_Na of Hep-High, and R = H or SO_3_Na, R1 = COCH_3_ or SO_3_Na, R2, R3 = H, COONa (n = 3~20).

Varying amounts of heparin were bound to BSF [31,32,33]. Two heparin types with different molecular weights were chosen: unfractionated, with an average molecular weight of 15,000 Da; and purified and reduced, with a molecular weight of 5000 Da. The reaction points of BSF and the molar ratio of each reagent used to prepare each sample are shown in Table 1. Unfractionated sodium heparin (Wako Pure Chemical Industries, Ltd., Osaka, Japan) and dalteparin sodium (Sigma-Aldrich, Saint Louis, MO, USA) were dissolved in 18 mL of Na_2_CO_3_/ultrapure water in pH 10.0 to 11.0. The scheme of the modified reaction process is shown in Figure 1. For the CY solution, 78 mg of 2,4,6-trichloro-1,3,5-triazine (CY, Kanto Chemical Co., Inc., Tokyo, Japan) was dissolved in 6.5 mL of 1,4-dioxane. While stirring each heparin aqueous solution under ice-cold conditions, 6.0 mL CY solution was added dropwise at a rate of 24 mL/h using a syringe pump. After 2 h of reaction, 24 mL of the BSF aqueous solution adjusted to 2.0 wt% was mixed with the heparin–CY solution, and the mixing was continued at 37 °C for 24 h. The reaction was stopped by adding 1 M hydrochloric acid to neutralize the solution and then dialyzing against ultrapure water. Dialysis was continued for more than 5 days, and fresh ultrapure water was used several times a day to remove unreacted substances and solvents. The dialyzed reaction solution was centrifuged at 4 °C, 8500 rpm for 30 min to remove the precipitate, and a heparin-modified silk fibroin (HSF) aqueous solution was obtained. As shown in Table 1, the samples are named HSF2, HSF3, HSF4, HSF5, HSF-High (HSFH), and HSF-Low (HSFL), depending on the molecular weight of the heparin used. 

The films used for each test were prepared as follows. The sponge obtained by freeze-drying each HSF aqueous solution was dissolved in formic acid and dried in a Teflon dish at room temperature. As an insolubilization treatment of the BSF, incubation was performed at 37 °C for 24 h under 100% relative humidity, then it was vacuum dried at room temperature. Scanning electron microscopy (SEM) images of the film surface were taken using VHX-D510 (Keyence Corp., Osaka, Japan). Film strips were applied to the tape and sputter coated with gold particles. Acceleration voltages of 0.9–1.2 kV were used.

### 2.3. HSF Reaction Confirmation

The progress of the heparin modification reaction was confirmed using solution-state ^1^H NMR spectroscopy [8,34]. Amounts of 500 μL of each HSF aqueous solution, the sodium heparin solution, and the BSF aqueous solution were prepared at a concentration of 0.6 wt% and mixed with 100 μL deuterated water. ^1^H NMR spectra of all samples were acquired on a JEOL ECA 500 spectrometer (JEOL Resonance, Tokyo, Japan) operating at 500 MHz for ^1^H observation using a 5 mm probe. A water pre-saturation (watergate pulse [wgh]) sequence and DPFGSE spectra were applied to all the samples. ^1^H NMR spectra were recorded with a 5 kHz spectral width and a 5.0 s relaxation delay. The amount of modification was calculated from a calibration curve obtained by plotting the intensity of the peak derived from the heparin acetyl-methyl group against BSF Ala H_β_ in the spectrum of a mixed sample of BSF and heparin.

### 2.4. Secondary Structure Analysis of Film

Solid-state NMR ^13^C CP/MAS NMR measurements examined changes in the BSF secondary structure in each HSF insolubilized film [35,36]. The ^13^C CP/MAS NMR method was used to measure the pulse sequence using an AVANCE WB 400 (Bruker, Japan K.K., Yokohama, Japan) at a spinning speed of 8.5 kHz and 4096 times accumulation with a resonance frequency of 400 MHz. The main acquisition parameters were as follows: contact time (5.00 ms), ^1^H 90° pulse (3.20 μs), and pulse delay (6.0 s). 

Fourier-transform infrared (FTIR) spectroscopy was performed using an FT/IR-4600 (JASCO Corp., Tokyo, Japan) with a single-reflection ZnSe ATR attachment (ATR PRO ONE, PKS-Z1, JASCO Corp., Tokyo, Japan). All spectra were recorded at 64 scans with a resolution of 4 cm^−1^. 

### 2.5. Evaluation of Film Chemical Properties

To investigate the change in the water characteristics on the film, static water contact angle (WCA) measurements were performed using a DMo-501Hi dispenser (Kyowa Interface Science Co., Ltd., Saitama, Japan); 2 μL of ultrapure water droplets were dropped onto each film, and the state of the droplets after 1 sec was photographed. The WCA with respect to the film surface was calculated using the θ/2 method. The study was repeated with independent HSF samples and films (n = 5).

The water content of the BSF and HSF film samples was measured to investigate the change in water absorption capacity. Film samples punched for 11 mm diameter were dried under reduced pressure at room temperature for 15 h or more, then their weights were measured as dry weight (Wd) [19]. The films were incubated in a dish with ultrapure water for 3 h at 37 °C. The weight of the films was measured again as wet weight (Ww). The swelling ratio was calculated from the following formula:

The swelling ratio (%) = [(Ww − Wd) / Ww] × 100(1)

The study was repeated with independent HSF samples and films (n = 10).

### 2.6. Dynamic Viscoelasticity Evaluation

Dynamic viscosity analysis (DVA) in the air and under water was performed using a DVA-205 (IT Metrology Control Co., Ltd., Osaka, Japan) to evaluate the effect of heparin modification on the mechanical properties reflected in the BSF crystallinity. Measurements in aerial conditions were performed using the tensile mode with a constant rate of temperature rise to calculate the average storage modulus, modulus loss, and loss coefficient at 37 ± 0.2 °C, a frequency of 1 Hz, and 30 mm distance between the gripper tools. Each film was cut into a rectangular shape of 40 mm × 5 mm. For the underwater measurements, films were cut into 25 mm × 5 mm and immersed in ultrapure water for 3 h. The mean storage modulus, modulus loss, and modulus of loss were calculated from the mean values measured for 10 min in the tensile mode. The measurement conditions were a water temperature of 37 ± 0.2 °C, a frequency of 1 Hz, and a distance between the gripper tools of 15 mm. Samples were taken from different independent films (n = 3).

### 2.7. Cell Culture

The HUVEC and Neo-NHDF lines (Lonza, Walkersville, MD, USA) were purchased and then cultured with the Clonetics™ pooled HUVEC-XL™ cell systems EBM™ 2 (Lonza), which contained endothelial basal medium, 2% (*v*/*v*) fetal bovine serum, 0.5 mL VEGF, 0.5 mL hEGF, 0.5 mL R3-IGF-1, 0.5 mL ascorbic acid, 0.2 mL hydrocortisone, 2 mL hFGF-β, 0.5 mL gentamicin/amphotericin-B, and 0.5 mL heparin. FBM-BulletKit™ (Lonza) contained fibroblast basal medium, 2% (*v*/*v*) fetal bovine serum, 0.5 mL hFGF-β, 0.5 mL insulin, and 0.5 mL gentamicin/amphotericin-B. Cell cultures were maintained in a humidified 5% CO_2_ atmosphere at 37 °C. In the HUVEC experiment, heparin was excluded from the kit to avoid cross-effects. The passage numbers used for the HUVECs were 3 and 4, and that for the NHDFs was 6.

### 2.8. Evaluation of Cell Proliferation

Films were prepared by cast-coating into wells in 24-well cell culture plates at 0.25 mg/cm^2^ and insolubilized. For sterilization, wells were soaked in 70% (*w*/*v*) ethanol for 20 min, washed twice with 1 × PBS, and then air dried. Before seeding, the wells were swollen with test growth medium.

NHDFs and HUVECs suspensions were prepared at 2.0 × 10^4^ cells/well and seeded with 500 μL of medium. Cells were cultured for 6 h, then gently washed with 1 × PBS to remove unattached cells. To evaluate metabolic activity, MTS reagents were added following the manufacturer’s protocol. Briefly, 40 μL of MTS reagent prepared with a volume ratio of MTS:PMS = 20:1 was added into 200 μL of fresh medium, then absorbance at 492 nm was measured after 2 h incubation. HUVECs and NHDFs were seeded at a density of 3.0 × 10^3^ cells/well. The mediums were replaced every 2~3 days. Depending on the healing mechanism, the NHDF cultures were maintained for up to 7 days longer than the HUVECs to investigate long-term growth behavior. The same assay was repeated 2~4 times using different HSF samples until the trend was confirmed to agree (n = 5).

### 2.9. Statistical Analysis

All data are presented as mean ± standard deviation. One-way ANOVA with Tukey’s statistical analysis was performed by multiple comparisons using Origin 2024 software (OriginLab, Northampton, MA, USA). A probability value (*p*) of less than 0.05 was considered statistically significant (* *p* < 0.05, ** *p* < 0.01).

## 3. Results

### 3.1. Reaction Verification

**Figure 2:**
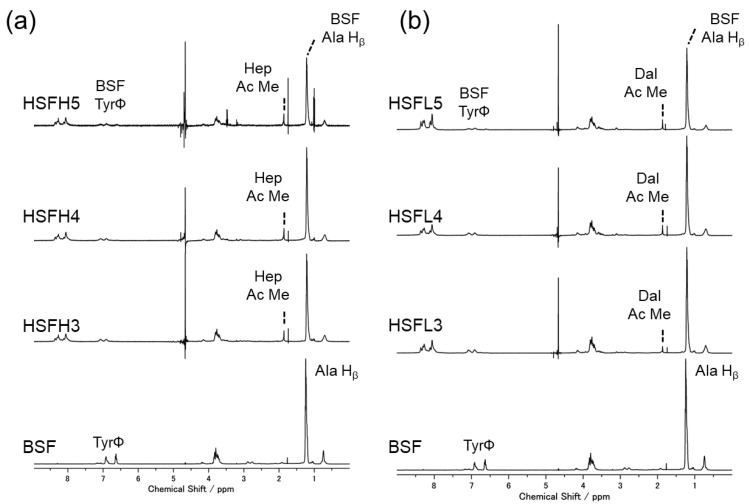
The spectrum of solution-state ^1^H NMR measurement: (**a**) HSFH, (**b**) HSFL. Amino acids of BSF and heparin acetyl-methyl derived peaks are marked.

All aqueous solutions of HSFs prepared by binding heparin to BSF were clear and colorless after dialysis. The obtained concentration was 0.5–1.0 wt%. Figure 2 shows the solution-state ^1^H NMR spectrum of each HSFH and HSFL solution compared with that of BSF. The characteristic peak derived from the alanine residue (H_β_) of BSF was close to 1.2 ppm, and that derived from the heparin acetyl group (CH_3_) was close to 1.9 ppm, which can be seen in all HSF spectra [8,37]. It was confirmed that heparin was present as bound to BSF in HSF collected after the removal of unreacted substrates through dialysis. Peaks derived from BSF Tyr residues appeared at 6.65 ppm and 6.93 ppm, but in the HSF sample, the intensity near 6.65 ppm significantly decreased, and a new peak appeared at 7.12 ppm. This change was consistent with our previous results reported for HSFH and was independent of the heparin molecular weight [8]. The chemical shift change was attributed to be due to changes in the local electron density of the Tyr aromatic hydrogen. Gotoh et al. also modified N-acetyl-d-glucosamine (GlcNAc), which is one of the constituent sugars of heparin, onto BSF Tyr residues also using CY and evaluated the progress of the reaction using ^1^H NMR measurements [32]. The Tyr OH group and C1 anomeric hydrogen of the sugars underwent a desorption–condensation reaction with CY, resulting in a low-field shift. From these spectra, it was confirmed that both substances were present in the heparin-bound samples. In addition, the enlarged area around the peak derived from heparin C-H shows that the amount of modification gradually increased for both samples with different heparin molecular weights. The ratio of the intensity of the Hep-High or Hep-Low acetyl-methyl group derived peaks to the intensity of Ala H_β_ in BSF was determined. The amount of modification was calculated from the calibration curve obtained by plotting the peak intensities against the heparin concentration using the same procedure with a sample of unreacted heparin powder mixed with BSF aqueous solution. Table 2 shows a stepwise increase in the amount of modified heparin in the HSF aqueous solution, independent of molecular weight. A slightly higher modification level was suggested for Hep-Low, which was modified with heparin of lower molecular weight. This may be due to the higher reactivity when the molecular weight is smaller.

### 3.2. Secondary Structure Analysis

**Figure 3:**
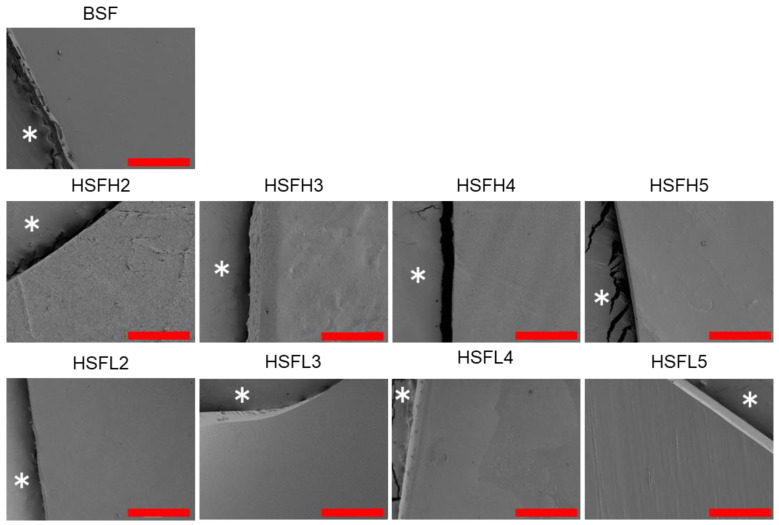
Scanning electron microscopy (SEM) images were taken around the edge of the film strips after insolubilization. *: tape, scale bar: 100 μm.

We prepared regenerated films by dissolving sponges obtained through lyophilization of BSF or HSF aqueous solutions in formic acid, followed by air-drying at room temperature. To enhance the water insolubility of BSF, a gentle reformation into β-sheet structures from random coils was induced as an insolubilization treatment under high-humidity conditions at 37 °C. SEM images near the edges of each film revealed consistently smooth surfaces (Figure 3). In blended cast films composed of synthetic polymers and/or natural polymers, phase separation called a sea-island structure is frequently observed [30]. However, distinct separation was not observed at the microscale, even though BSF consisting of hydrophobic crystals was modified with hydrophilic and bulky polysaccharides. As indicated by some images, the cross-sectional view of the films revealed densely packed, smooth surfaces, suggesting uniformity throughout the entire film.

**Figure 4:**
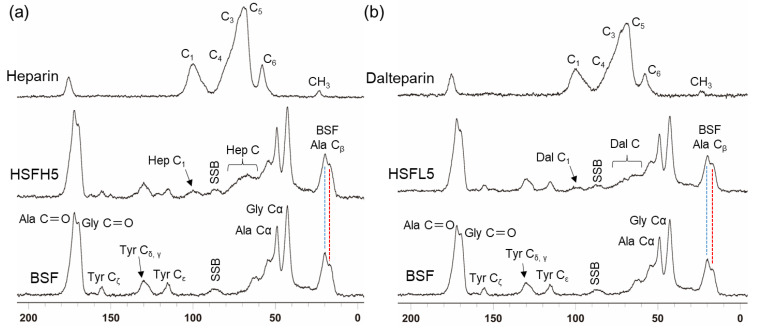
The spectrum of solid-state ^13^C CP/MAS NMR measurement at 25 °C. BSF and (**a**) HSFH5 or (**b**) HSFL5, normalized by the intensity of the Ala C_β_ peak in the BSF. For the peak derived from Ala C_β_, the random coil shows the peak at 16.8 ppm (red line) and the β-sheet structure shows the peak at 19.8 ppm (blue line). All films were insolubilized.

Figure 4 displays the spectra obtained by measuring the solid ^13^C CP/MAS NMR spectra of the HSF film after insolubilization treatment. Of these spectra, particular attention was given to the peaks derived from the alanine residue C_β_, which appear between 10 and 30 ppm and reflect the secondary structure of BSF. In the insolubilized BSF spectrum, the 19.8 ppm peak showing the β-sheet structure appeared more predominantly than the 16.8 ppm peak of the random coil [38]. This means that even in HSF5, which has the highest amount of modification, the β-sheet structure achieved a dominant hydrophobic crystal reformation. Waveform deconvolution of Ala C_β_-derived peaks is commonly used as a quantitative analysis of BSF crystallinity (16.6 ppm: random coil and repeated β-turn; 19.6 ppm and 21.9 ppm: β-sheet structure) [39]. However, both polysaccharides exhibited a methyl group derived broad peak around 23 ppm. Even for low-molecular-weight dalteparin, the large number of repeats of the constituent sugars and long relaxation times made it difficult to combine another 2D and DP/MAS measurement. The intensity of the polysaccharide-derived peak in HSF was slightly higher at HSFH but did not reflect the difference in the molecular weight. The CP/MAS measurement, which can obtain spectra that emphasize relatively lower kinetic components, may have resulted in lower intensities of polysaccharide-derived peaks of lower molecular weight than in BSF.

**Figure 5:**
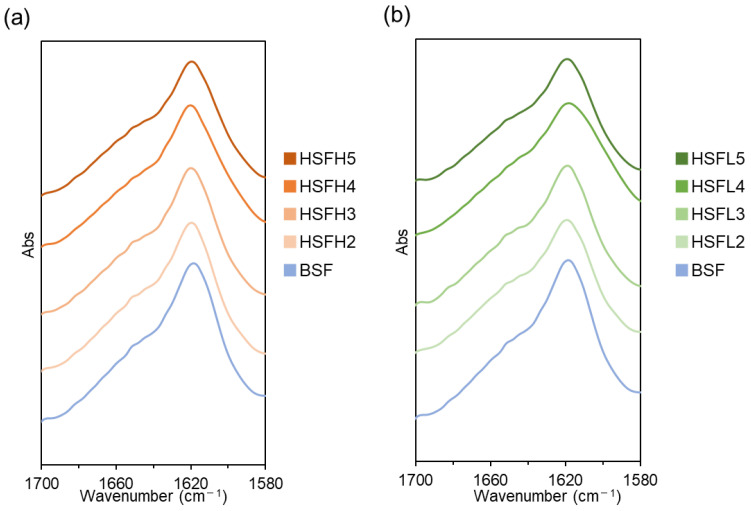
ATR-FTIR spectra in the Amide I region for BSF, (**a**) HSFH and (**b**) HSFL films after insolubilization treatment.

The ATR-FTIR spectra of the BSF and each HSF film in the Amide I region are presented in Figure 5. In both HSFH and HSFL, spectra closely resembling BSF, with peaks around 1618 cm^−1^, were obtained. FTIR measurement of BSF is a frequently employed technique due to its simplicity and the nondestructive nature of the material. It also allows for a more detailed discussion of the crystalline structure by separating the Amide I peak into peaks derived from each secondary structure. As summarized by Hu et al., peaks assigned to the β-sheet structure are typically found in the range of 1616–1637 cm^−1^ [40]. In BSF, the maximum was observed at 1617 cm^−1^, while in the HSFs, it ranged from 1617 to 1618 cm^−1^. Additionally, peaks attributed to random coil structures were assigned to two regions: 1638–1646 cm^−1^ and 1647–1655 cm^−1^. For the lower range, a shoulder peak at 1638 cm^−1^ was detected in both the BSF and all HSF samples. BSF exhibited a shoulder peak at 1650 cm^−1^, while in HSF, another shoulder peak was observed in the range of 1650–1651 cm^−1^ The positioning of the maximum of the Amide I peak in the region assigned the β-sheet structure indicates that the prepared HSF films achieved the reformation of β-sheet crystalline structures similar to BSF films. However, the quantitative evaluation of the crystalline structure through waveform separation of the Amide I peak was also challenging. The Amide I peak reflects the C=O stretching of peptide bonds, and both heparin and dalteparin had C=O bonds in this region. The ATR-FTIR measurements used in this study are a local secondary structure analysis method near the film surface. Multiple points were measured for the same film, and waveform separation was performed under the same conditions. While the spectral shapes and peak frequencies matched, determining the ratios of polysaccharide-derived peak components proved difficult. Through the combined use of both solid-state NMR measurements and FTIR measurements, the qualitative observation of the presence of reformed β-sheet structures in HSF films was possible. The presence of hydrophobic crystalline structures in HSF films, based on the characteristics of BSF, suggested the acquisition of water insolubility and mechanical properties.

### 3.3. Chemical Properties of Film and Film Surface

**Figure 6:**
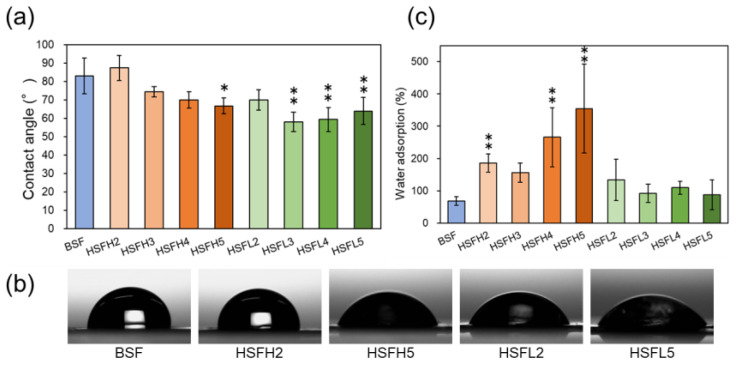
(**a**) The static water contact angle (WCA) on each BSF and HSF film (n = 5). (**b**) Representative images of the WCA. (**c**) The content of water adsorption of BSF and HSF films (n = 10). *: *p* < 0.05 and **: *p* < 0.01 compared with BSF as a control.

The hydrophilicity of the film surface was evaluated on the basis of its static water contact angle (Figure 6a,b). As the amount of modification increased, the hydrophilicity of the HSFH gradually improved. The hydrophilicity value of all HSFL was as high as that of HSFH5, regardless of the increase in the amount of modification. The reason for this could be the exposure of heparin on the film surface, and the dalteparin might have achieved equilibrium due to its higher density and superficial appearance. Both approached about 60–70 degrees, which is suitable for the initial adhesion of cells [41]. 

Figure 6c shows the moisture content calculated from the wet weight ratio and dry weight of each HSF and BSF film. As shown in these results, HSFH initially exhibited a gradual improvement in water content, followed by a drastic improvement. On the other hand, that of HSFL was similar, with slightly improved swelling ability, although it was higher than that of BSF. This tendency is similar to that of the contact angle, and we verified that the water characteristics of BSF are strongly influenced by the molecular weight of heparin.

### 3.4. Dynamic Viscoelasticity Evaluation

**Figure 7:**
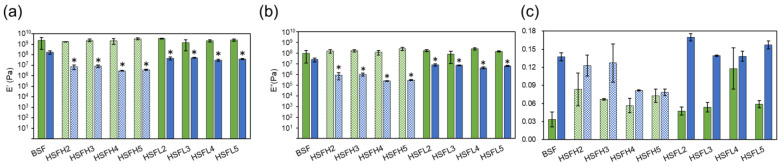
(**a**) The storage elastic modulus (E’), (**b**) loss elastic modulus (E”), and (**c**) the loss factor (tan δ) of BSF and HSF films. Green bars show the value measured in the aerial state and blue bars show the value in the underwater state at 37 °C. *: *p* < 0.05 compared with BSF as a control.

Dynamic viscoelasticity measurements were conducted in air and water to evaluate the storage elastic modulus (E’), loss modulus (E”), and loss coefficient (tan δ) of the HSF and BSF films (Figure 7). Under aerial conditions, the BSF and HSF groups exhibited similar elastic moduli, regardless of the modified amount and heparin molecular weight. However, in underwater conditions, both HSF groups showed lower elastic moduli than BSF. Additionally, a tendency similar to the swelling ratio was observed among the HSFs, particularly HSFH films, which showed a modification-dependent decrease in the elastic modulus. Moreover, the calculated loss coefficients indicated that both the BSF and HSF groups had stronger viscous properties underwater than in aerial conditions, possibly indicating the presence of water molecules inside the material. It was indicated that HSFL exhibited a higher loss coefficient than SF in underwater conditions, while HSFH showed a lower one. This suggests that the water molecules inside each material had a different interaction state from those between the materials when the molecular weight of heparin varied. It has been reported that the interaction of water molecules with the material differs from that of bulk water [42]. The type and quantity of serum protein adsorbed on the material surface undergo changes influenced by water molecules [43,44,45], serving as one of the factors affecting the cellular response.

### 3.5. Cell Proliferation In Vitro

**Figure 8:**
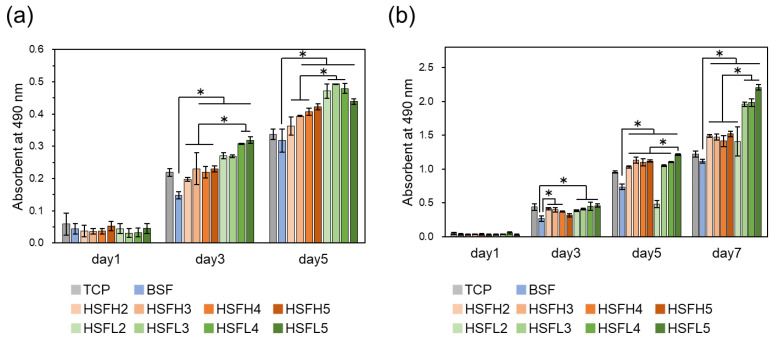
The proliferation of (**a**) HUVECs and (**b**) NHDFs on cast-coated films of BSF and HSF. Non-coated well (TCP) was used as a control group evaluated with MTS assay. For day 1, there was no significant difference in either graph. *: *p* < 0.05.

This experiment aimed to gain fundamental insights into the impact of material chemical properties such as hydrophilicity and the capture and provision of GFs on cell proliferation. Thin-film coating on cell culture plates was chosen to eliminate the influence of film stiffness. In addition, growth mediums containing serum and various GFs were used in the experiments. In both NHDFs and HUVECs, cell proliferation in the HSF group was significantly enhanced compared with BSF on the third day of culture, and this promotion increased with extended culture duration (Figure 8). Finally, for both cell types, cell proliferation was significantly promoted when the amount of heparin modification was HSF3 or higher and the HSFL value was significantly higher than that of HSFH. The result that HSFL more strongly promoted cell proliferation was contrary to the results of preliminary experiments; the amount of GF capture was dependent on the molecular weight of heparin [26]. In this experimental system, we evaluated the proliferation behavior of cells adhered to the surface of the coating film. This proliferation could be due to (1) higher exposure on the film surface and (2) increased ease of interaction with GFs due to decreased structural heterogeneity, potentially enhancing the establishment of interactions between cells and GFs. There was concern that reducing the molecular weight of heparin could reduce the cell proliferative capacity due to a decrease in the sites of interaction with GFs. Interestingly, it yielded superior results when molecular weight was decreased, contrary to expectations. Regarding the modification amount, a trend of higher values was observed for HSF3 and above. HSFs were expected to provide an excellent foundation for various cells involved in tissue regeneration, inducing tissue renewal through the promotion of cell activity [46].

## 4. Discussion

This study fabricated functionalized BSF materials with improved cell responsiveness by immobilizing heparin via chemical modification. We prepared multiple samples with different molecular weights and weight ratios of the heparin used in the reaction. In previous studies, the effects of modified reactions on the secondary structure transfer, physics, and cellular responsiveness of BSF have been widely evaluated for the most basic form of film samples. The BSF is roughly divided into a crystalline region composed of a repeating sequence of (GAGAGS)_n_ and a non-crystalline region containing a hydrophobic quasicrystal sequence of ((GX)_m_GY)_n_ (X = A or V) and amorphous [20]. When placed in an organic solvent or under high-humidity conditions, a tightly packed crystal structure with a β-sheet structure is induced around the crystal region, which acquires the toughness and water insolubility specific to BSF [47,48,49]. To date, studies have reported the chemical modification of functional molecules in BSF. However, the modification of high-molecular-weight, highly hydrophilic polysaccharides such as heparin disturbs the formation of this characteristic crystal structure. It is considered to be due to steric hindrance. A material fabrication method was chosen to mitigate the impact of polysaccharide modification on the crystalline structure reformation of BSF, aiming to avoid steric hindrance and hydrophilicity mismatch. This involved (1) setting the reaction points on the BSF side to tyrosine residues present in the non-crystalline region; (2) changing the casting solvent from water to formic acid, which induces β-sheet structure; and (3) gently inducing secondary structure transition and realignment under high-humidity conditions [50]. 

In the regenerated films prepared with formic acid as the solvent, structural transitions occurred regardless of the molecular weight of heparin. Both films exhibited a dominant secondary structure of β sheets indicating the prevalence of the β-sheet structure over random coils, as revealed with the ATR-FTIR measurements and solid-state ^13^C CP/MAS NMR measurements. In other words, it was qualitatively confirmed that the BSF component in the HSF underwent reformation of the crystalline structure. To evaluate the crystalline structure of BSF further quantitatively, it is possible to separate the peak components derived from each secondary structure and compare the transitions in their respective proportions [38,51]. However, when using the polysaccharide heparin, the spectrum overlaps with the protein BSF. In such cases, it is sometimes effective to separate them by measuring solid-state NMR with stable isotope labeling, such as [1-^13^C] Ala and [1-^13^C] Gly [52]. However, labeling on the sugar side is not practical due to the structural heterogeneity and the difficulty of labeling. While it is relatively straightforward for BSF, using a simple method involving feeding larvae, securing an adequate sample amount remains a challenge [53]. Therefore, we tried to indirectly evaluate the structure through physical property assessments rather than attempting structural evaluation directly.

The modified amounts of HSFH and HSFL with different molecular weights were estimated in moles and were found to be comparable to the introduction rate. The heparin content introduced was approximately 7.0–10.1 mol% for Hep-High and 8.2–11.8 mol% for Hep-Low. In the case of N-Acetyl-chito-oligosaccharides (NACOS)-BSF modified with oligosaccharides consisting of 3–4 repeats of GlcNAc, the ratio of BSF:NACOS-BSF was 0.8:1, resulting in an estimated modification of around 17% [32]. Considering that the molecular weights of the heparins used differed by approximately threefold, it was speculated that the reaction efficiency was significantly reduced for the monosaccharides due to steric hindrance. However, there was a large gap in the hydrophilicity and water content of the material surface, even when the amount of modification was the same.

The water contact angle (WCA) gradually decreased in HSFH, reflecting the hydrophilicity of exposed heparin on the film surface. On the other hand, HSFL showed values similar to HSFH5, with a tendency to be lower, especially in HSF3-5. The water content increased proportionally with the modification amount in HSFH; although it was slightly higher than BSF, the values among HSFL were similar. Considering these results, it might be suspected that the fine structure of the material’s interior capable of incorporating water molecules is different. The hydrophobic crystalline structure of BSF is densely packed with β-sheet structures. Therefore, heparin molecules bound to Tyr residues in the amorphous region surrounding this crystal domain are believed to be scattered in the hydrophobic domain. In HSFH, containing a larger molecular weight of heparin, the spaces among these crystalline structures would likely increase due to steric hindrance, allowing for the inclusion of more water molecules. The amount of exposure to the film surface is expected to be higher in HSFL, which takes smaller aggregated structures per unit area. In fact, when PEG is modified on the material surface, it has been reported that using longer linear PEG molecules leads to a more bulky and aggregated presence [54]. Furthermore, the results of DMA measurements supported the differences in the state of the water molecules in HSFH. In other words, interactions between the material and water molecule implied that the molecular weight of heparin affected the 3D network inside the material. The heparin, which coexisted in the amorphous region where BSF crystals were scattered, strongly interacted with water molecules due to its charge; however, this effect was reduced by decreasing the molecular weight, which simplifies the structure. The presence of intermediate water molecules (loosely bound water), which undergo low-temperature crystallization and melting below 0 °C due to interactions with the material, has been reported in heparin [55].

Cell tests showed that both HSFH and HSFL significantly promoted the adhesion and proliferation of endothelial cells and fibroblasts compared with BSF. Additionally, the greatest growth of the HSF group with increasing culture time may have been because of GF trapped in the material, which was significantly increased by changes in the heparin molecular weight. Because the cells were adherent to the film surface, the amount of GF trapped in heparin exposed on the film surface may have had a stronger effect on cell growth enhancement than the amount retained throughout the film. The function of GF in structural stabilization has not been fully elucidated, but it is believed that structural differences at the molecular level have an effect, as in the model presented here. These factors may affect long-term GF storage in vivo, demonstrating the need for further analysis using solid-state NMR and hydrous DSC measurements [42,56].

## 5. Conclusions

Material modification through the introduction of functional molecules is widely used in the development of tissue-engineering materials. Although naturally derived polymers are chosen for their biocompatibility and advantages in inducing tissue regeneration, their heterogeneous chemical structures often make structural analysis difficult and reduce the reproducibility of the materials. In this study, we developed a scaffold based on silk fibroin from the domesticated silkworm, BSF, chemically modified with heparin, a component of the extracellular matrix. The hydrophobic crystal structure, composed of the characteristic β-sheet structure of BSF, was regenerated as disordered crystals whose orderliness was reduced by the heparin modification. Therefore, from the comparison of BSF materials prepared by modifying unfractionated heparin of biological origin and low-molecular-weight dalteparin by the same procedure, we discussed the influence of molecular weight and modification amount as parameters on the material properties. The film wettability and cell-growth-promoting capacity were significantly improved in the dalteparin group compared with the heparin group; the effect of steric hindrance on the remodeling of the BSF crystal structure was suppressed, thus maintaining the mechanical strength in the hydrated state. It was feared that the reduction in growth factor interaction sites due to the low molecular weight of heparin would reduce the degree of cell proliferative potential. The fact that the dalteparin group was highly effective in promoting the proliferation of skin-regeneration-related cells is an example of how naturally derived polymers can be selected for use after low molecular weight and purification without compromising their natural function. The results of this study suggest that the amount of HSF3 to HSF4 (i.e., a little less than 10 mol%) was sufficient to achieve both functional and mechanical properties. This can be extended to material design using other biogenic polymers by considering the amount of exposure on the material surface and the balance of the multi-component material structure. In this study, we demonstrated the utility of reducing the molecular weight of polysaccharides to adjust the balance between improving cell affinity and preserving mechanical properties in heparin-modified silk fibroin. This allowed us to reconcile enhancing cell affinity while maintaining mechanical characteristics through the stability of the material. As a preliminary step in exploring processing conditions for various forms like nanofiber sheets and sponges, it provides a material design strategy for modification using chemical reactions.

## Figures and Tables

**Table 1:** The molar ratio of each reagent used for the chemical reaction of each HSF.

	Target Sites in BSF (mol)	Cyanuric Chloride (mol)	Heparin Sodium (mol)
HSFH2, HSFL2	20	20	2
HSFH3, HSFL3	20	20	3
HSFH4, HSFL4	20	20	4
HSFH5, HSFL5	20	20	5

**Table 2:** The ratio of the molecular amount of heparin in each HSF solution.

	HSFH (mol%)	HSFL (mol%)
HSF2	7.03	8.19
HSF3	7.16	8.35
HSF4	8.65	10.07
HSF5	10.10	11.77

## Data Availability

Data are contained within the article.
